# Regulation Mechanism Mediated by *Trans*-Encoded sRNA Nc117 in Short Chain Alcohols Tolerance in *Synechocystis* sp. PCC 6803

**DOI:** 10.3389/fmicb.2018.00863

**Published:** 2018-05-01

**Authors:** Yanqi Bi, Guangsheng Pei, Tao Sun, Zixi Chen, Lei Chen, Weiwen Zhang

**Affiliations:** ^1^Laboratory of Synthetic Microbiology, School of Chemical Engineering and Technology, Tianjin University, Tianjin, China; ^2^Key Laboratory of Systems Bioengineering, Ministry of Education of the People’s Republic of China, Tianjin, China; ^3^Collaborative Innovation Center of Chemical Science and Engineering, Tianjin, China; ^4^Center for Biosafety Research and Strategy, Tianjin University, Tianjin, China

**Keywords:** *trans*-encoded sRNA, *Synechocystis*, tolerance, biofuels, regulation

## Abstract

Microbial small RNAs (sRNAs) play essential roles against many stress conditions in cyanobacteria. However, little is known on their regulatory mechanisms on biofuels tolerance. In our previous sRNA analysis, a *trans*-encoded sRNA Nc117 was found involved in the tolerance to ethanol and 1-butanol in *Synechocystis* sp. PCC 6803. However, its functional mechanism is yet to be determined. In this study, functional characterization of sRNA Nc117 was performed. Briefly, the exact length of the *trans*-encoded sRNA Nc117 was determined to be 102 nucleotides using 3′ RACE, and the positive regulation of Nc117 on short chain alcohols tolerance was further confirmed. Then, computational target prediction and transcriptomic analysis were integrated to explore the potential targets of Nc117. A total of 119 up-regulated and 116 down-regulated genes were identified in *nc117* overexpression strain compared with the wild type by comparative transcriptomic analysis, among which the upstream regions of five genes were overlapped with those predicted by computational target approach. Based on the phenotype analysis of gene deletion and overexpression strains under short chain alcohols stress, one gene *slr0007* encoding D-glycero-alpha-D-manno-heptose 1-phosphate guanylyltransferase was determined as a potential target of Nc117, suggesting that the synthesis of LPS or S-layer glycoprotein may be responsible for the tolerance enhancement. As the first reported *trans*-encoded sRNA positively regulating biofuels tolerance in cyanobacteria, this study not only provided evidence for a new regulatory mechanism of *trans*-encoded sRNA in cyanobacteria, but also valuable information for rational construction of high-tolerant cyanobacterial chassis.

## Introduction

Biofuels have become a hot research area in recent decades due to their potential of replacing fossil (Atsumi et al., 2008). Cyanobacteria, as a group of autotrophic prokaryotes with the advantages of rapid growth, only consuming solar, water and CO_2_, hold great promise as an important alternative to generate biofuels (Angermayr et al., 2009). In recent years, cyanobacteria have been successfully engineered to synthesize various types of biofuels (Gao X. et al., 2016). For example, Lan et al. (2013) successfully incorporated biofuels biosynthetic pathways into cyanobacterial systems, and 404 mg/L production of 1-butanol was achieved in *Synechococcus elongatus* PCC 7942 (Lan et al., 2013). In addition, Gao Z. et al. (2016) reached 5.5 g/L production of ethanol by systematically optimizing metabolic pathway for ethanol production in engineered cyanobacteria (Gao Z. et al., 2016). However, there is still a big gap between the current productivity in cyanobacteria and other engineered bacteria, such as *Escherichia coli* and *Clostridium*, and the low tolerance to biofuels toxicity of cyanobacteria is regarded as one of the important limiting factors (Jin et al., 2014). It was found that the toxic effects caused by the product ethanol or isobutanol would result in significant cell growth retardation in *Synechocystis* sp. PCC 6803 (hereafter *Synechocystis*) and *S. elongatus* PCC 7942 (Atsumi et al., 2009; Dexter and Fu, 2009), thus limiting the potential of these biofuels production and the possibility of industrial application. In another study, it was reported that the addition of 0.20% (*v*/*v*) butanol caused a 50% growth decrease in *Synechocystis* (Tian et al., 2013), suggesting the great toxicity caused by biofuel butanol. Therefore, resistance mechanism of cyanobacteria to biofuels needs to be investigated, which is an important prerequisite for constructing highly biofuels-tolerant strains to improve the biofuels production in cyanobacteria. Recently, Kaczmarzyk et al. (2014) by overexpressing an RNA polymerase sigma factor, successfully increased butanol tolerance and lowered the intracellular concentration of reactive oxygen species in *Synechocystis*. Moreover, in our previous work, several regulatory genes (i.e., *slr1037, sll0039, sll0794*, and *slr1860*) related to alcohols tolerance were successfully identified (Song et al., 2014; Zhu et al., 2014; Gao et al., 2017). For the resistance mechanism to biofuels, based on several previous studies, cyanobacteria employed a combination of multiple resistance systems to adapt to biofuels stress or nutrient limited environments, including cell membrane tolerance mechanism, transport vector system, intracellular transformation and degradation, cell surface structure and morphology changes, as well as common pressure response tolerance mechanisms (Jie et al., 2012; Qiao et al., 2012, 2013; Huang et al., 2013; Tian et al., 2013; Xu et al., 2014). However, this brings great challenges to further improve tolerance by conventional sequential multi-gene modification approaches (Gao X. et al., 2016). As an alternative, “transcriptional engineering” for tolerance improvement (Anders and Huber, 2010), especially the sRNAs engineering that has the advantages such as rapid response, low metabolic burden and flexible and precise control, could be an applicable approach (Gaida et al., 2013).

Bacterial sRNAs between 50 and 300 nucleotides transcribed from the intergenic region, play important regulatory roles at the levels of post-transcriptional, regulating translation, decaying or protecting mRNA molecules, through base paring in bacterial cells (Johansen et al., 2008; McCullen et al., 2010). To some extent, deciphering sRNAs regulatory mechanism is still challenging due to their functional complexity. For example, sRNAs as a kind of regulatory molecules, mediate cellular responses to the environment stress, not by means of encoding protein products, but via the complementary pairs with mRNA molecules or via combining with some protein to influence molecular function of protein activity (Kopf and Hess, 2015). Base-pairing between targets of sRNA and mRNA may cause inhibition or activation of mRNA translation, or affect the stability of the target RNA by promoting or blocking cleavage of a ribonuclease (RNase) (Saramago et al., 2014). Besides, sRNA may interact with RNA chaperone protein Hfq to modify its activity and then regulate gene expression of a diversity of targets (Updegrove et al., 2016).

Although some of sRNAs have been systematically investigated in cyanobacteria, e.g., IsrR involved in iron depletion (Duhring et al., 2006), As1-Flv4 involved in inorganic carbon supply (Eisenhut et al., 2012), PsbA2R, PsbA3R (Sakurai et al., 2012), PsrR1 (Georg et al., 2014), and RblR (Hu et al., 2017) related to photosynthetic gene expression, and NsiR4 (Klahn et al., 2015) controlling nitrogen assimilation, several systematic sRNA studies suggested there were thousands of sRNAs in cyanobacteria (Mitschke et al., 2011; Kopf et al., 2014; Xu et al., 2014), and the majority of them were still functionally uncharacterized. It is noteworthy that based on our previous work, a *trans*-encoded sRNA Nc117 involved in tolerance to exogenous ethanol and 1-butanol was identified in *Synechocystis* (Pei et al., 2017). Due to the complexity of sRNA regulatory mechanism, details about this *trans*-encoded sRNA Nc117 involved in the biofuels tolerance are still unclear. To determine potential targets of Nc117 sRNA, in this study, a transcriptomic analysis combined with computational target prediction were utilized to explore its potential targets, and one target gene *slr0007* encoding HddC positively regulated by Nc117, which participated in the tolerance to short chain alcohols in *Synechocystis* was successfully identified. As the first study of *trans*-sRNA involving biofuels tolerance in cyanobacteria, this study provided not only novel insights in regulatory mechanisms of sRNAs, but also a valuable target for biofuels tolerance enhancement in cyanobacteria.

## Materials and Methods

### *Synechocystis* Culture Conditions

For *Synechocystis* sp. PCC 6803 (ATCC 27184), WT and mutants were grown in BG11 medium (pH 7.5) under a light intensity of approximately 50 μmol photons m^-2^ s^-1^ in an illuminating incubator (HNY-211B Illuminating Shaker, Honour, China) at 130 rpm and 30°C with a starting cell density of OD_630_ = 0.04 (Pei et al., 2017). Cell density was measured with an ELx808 Absorbance Microplate Reader (BioTek, Winooski, VT, United States) at OD_630_. Five mL fresh cells at OD_630_ of 0.2 were collected by centrifugation at 3000 × *g* and 4°C and were then inoculated into 25 mL of BG11 liquid medium in a 100-mL flask. Ethanol 1.5% (*v*/*v*) and 1-butanol 0.25% (*v*/*v*) were added at the beginning of cultivation. 200 μL of culture was sampled and measured at OD_630_ every 24 h. Growth experiments were repeated at least three times to confirm growth patterns.

### 3′ RACE Analysis

Total RNA of *Synechocystis* was extracted under identical condition with transcriptome analysis (See below). Then RNA was added with a poly(A) tail using NEB *E. coli* poly(A) polymerase (New England Biolabs Inc., Ipswich, MA, United States). After that a specific primer containing oligo dT: 5′-CGTTGTAAAACGACGGCCAGTTTTTTTTTTTTTTTTTT-3′ and SuperScript^®^ VILO^TM^ cDNA Synthesis Kit were used to synthesize cDNA. At last, forward primer (5′-CGTCCAAACCTGAATAGATAATCCAT-3′) and reverse primer (5′-CGTTGTAAAACGACGGCCAG-3′) were used to amplify Nc117 containing 3′ end. The PCR product was purified and ligated into pTZ57R/T using InsTAclone PCR Cloning Kit (Thermo Fisher Scientific Inc., MA, United States) for sequencing analysis.

### Mutants Construction

For construction of gene overexpression strains, *E. coli* DH5α was used for vectors construction and enrichment. Primers used in this study were listed in **Table 1**. Gene expression vector pJA2, kindly provided by Prof. Paul Hudson (KTH Royal Institute of Technology of Sweden) (Huang et al., 2010; Kaczmarzyk et al., 2014), was used to overexpress the sRNAs or target genes. All sRNAs or genes were cloned under the control of the *psbA2* promoter. Briefly, the pJA2 backbone was amplified by PCR, treated with *Dpn*I and digested with *Bam*HI and *Xba*I to create cohesive ends. The sRNA or genes sequence was PCR-amplified using primers pJA2-*sRNA/gene*-F and pJA2-*sRNA/gene*-R and cloned into the *Bam*HI/*Xba*I sites of pJA2, resulting in the recombinant plasmid pJA2-*sRNA/gene*. The plasmid was introduced into the WT by electro-transformation as previously described (Wang et al., 2016). Positive clones were grown on BG-11 agar plates with 10 μg/mL kanamycin and were confirmed by colony PCR analysis.

**Table 1:** Primers used for mutants construction.

Primer names	Primer sequences (5′ to 3′)
pJA2-*nc117*-F	TGCTCTAGAAACTTAAGAGCGAAGTAAGT
pJA2-*nc117*-R	CGCGGATCCGAAAATGGAAAGAAGACGCT
Δ*nc117*-F1	ACCCCCGATGATTTTGCCAT
Δ*nc117*-R1	CCAGTGGCTTCTGTTTCTATCAGCTTTCGCTCTTAAGTTCATGCC
Δ*nc117*-F2	AGCTGATAGAAACAGAAGCCACTGG
Δ*nc117*-R2	TTACGCCCCGCCCTGCCACTCATCG
Δ*nc117*-F3	CGATGAGTGGCAGGGCGGGGCGTAAATACTTTCCGAGCCCAGAAT
Δ*nc117*-R3	AATAGCTGTCGGCGATGGAG
pJA2-*slr0007*-F	TGCTCTAGAATGGCTCTTTCCCCCGCAGA
pJA2-*slr0007*-R	CGCGGATCCCTATTTGTCTAGGTCTTGAA
Δ*slr0007*-F1	CCCTGCCATTCAATCCGTCT
Δ*slr0007*-R1	CCAGTGGCTTCTGTTTCTATCAGCTAGAGTTGAAAAAGTAGAAAC
Δ*slr0007*-F2	AGCTGATAGAAACAGAAGCCACTGG
Δ*slr0007*-R2	TTACGCCCCGCCCTGCCACTCATCG
Δ*slr0007*-F3	CGATGAGTGGCAGGGCGGGGCGTAACTTAAAATGAGAAGCTAACT
Δ*slr0007*-R3	CAAGTTGATGCAGAGCGTGG
pJA2-*slr2126*-F	TGCTCTAGAATGTTTTTCATGCAAAATACTAAGT
pJA2-*slr2126*-R	CGCGGATCCTCACAAAGTTAGTTTTTGATCGAGA
Δ*slr2126*-F1	TTGTAATGGCCCCAGCTTGT
Δ*slr2126*-R1	AGCTGATAGAAACAGAAGCCACTGGGAATTAATAATATTCTTTGT
Δ*slr2126*-F2	AGCTGATAGAAACAGAAGCCACTGG
Δ*slr2126*-R2	TTACGCCCCGCCCTGCCACTCATCG
Δ*slr2126*-F3	CGATGAGTGGCAGGGCGGGGCGTAATACCCCCTACTACAAATCCC
Δ*slr2126*-R3	TCCGCTTCTTGGGACTGTTC
pJA2-*sll1830*-F	TGCTCTAGAATGATCAATCGTCAGGACCT
pJA2-*sll1830*-R	CGCGGATCCTCAGTAGCGTAAAACCAAGG
Δ*sll1830*-F1	CTTTCCATGGCCGCTAAACG
Δ*sll1830*-R1	CCAGTGGCTTCTGTTTCTATCAGCTGGCCATTGATTAAGTTAAGG
Δ*sll1830*-F2	AGCTGATAGAAACAGAAGCCACTGG
Δ*sll1830*-R2	TTACGCCCCGCCCTGCCACTCATCG
Δ*sll1830*-F3	CGATGAGTGGCAGGGCGGGGCGTAATTTGTCTTTACATAGGTCGA
Δ*sll1830*-R3	CCCGAATTTCTGTGCTCCCA
pJA2-*slr2108*-F	TGCTCTAGAATGGGAGTGGATGGGATGAC
pJA2-*slr2108*-R	CGCGGATCCTTAGGTCGTACATAAGTGCC
Δ*slr1501*-F1	GCATTGGGCAGTTGTAAGCC
Δ*slr1501*-R1	CCAGTGGCTTCTGTTTCTATCAGCTGACCCCTTGGGGAAAGTTTT
Δ*slr1501*-F2	AGCTGATAGAAACAGAAGCCACTGG
Δ*slr1501*-R2	TTACGCCCCGCCCTGCCACTCATCG
Δ*slr1501*-F3	CGATGAGTGGCAGGGCGGGGCGTAAAAAAGATAACTACCATGTTT
Δ*slr1501*-R3	ACCTAGTTCCATCACCCCGA
Δ*sll0784*-F1	TCGGCTTTTCCGGGCATAAT
Δ*sll0784*-R1	CCAGTGGCTTCTGTTTCTATCAGCTGTACAAAAATTTAAGGATTA
Δ*sll0784*-F2	AGCTGATAGAAACAGAAGCCACTGG
Δ*sll0784*-R2	TTACGCCCCGCCCTGCCACTCATCG
Δ*sll0784*-F3	CGATGAGTGGCAGGGCGGGGCGTAAAACGACTGGAATAGTTTCAG
Δ*sll0784*-R3	AAGGAAACTTCGCTTCTACATTGA
pJA2-up500-F	GTTCCGCGCACATTTCCCCGA

For gene knockout and complementation mutants construction, a fusion PCR-based method was employed according to previous study (Wang et al., 2002). Briefly, for the gene target selected, three sets of primers were designed to amplify a linear DNA fragment containing the chloramphenicol resistance cassette (amplified from a plasmid pACYC184) with two flanking arms of DNA upstream and downstream of the target gene. The linear fused PCR amplicon was used directly for transformation into WT or WT-pJA2-*nc117* strain by natural transformation. The chloramphenicol-resistant transformants were obtained and passed several times on fresh BG11 plates supplemented with 10 μg/mL chloramphenicol to achieve complete chromosome segregation (confirmed by PCR). The successful knockout mutants were confirmed by PCR and sequencing analysis. PCR primers for mutant construction were listed in **Table 1**.

### Computational Prediction of sRNA Targets

After determination of the end of *trans*-encoded sRNA Nc117, target prediction was performed by IntaRNA software (Busch et al., 2008), and only the top 100 predictions obtained from IntaRNA with a free-energy cut-off of -15 kcal/mol were retained to remove potential false positive targets.

### RNA Samples Collection and Library Preparation for Transcriptomic Analysis

The WT and Nc117 overexpression strains of *Synechocystis* were cultured under same condition as previous growth comparison, each sample with two biological replicates for RNA collection. Approximately 10 mg of cell pellets were frozen in liquid nitrogen immediately after centrifugation at 8000 × *g* for 10 min at 4°C, and cell walls were broken by liquid nitrogen mortar grinding. Total RNA extraction was achieved using a miRNeasy Mini Kit (Qiagen, CA, United States). Contaminating DNA in RNA samples was removed with DNase I according to the instructions for the miRNeasy Mini Kit (Qiagen, CA, United States). Total RNA of each sample was quantified and qualified by Agilent 2100 Bioanalyzer (Agilent Technologies, Santa Clara, CA, United States), NanoDrop (Thermo Fisher Scientific Inc.) and 1% agarose gel. For each sample, 3 μg total RNA with RNA integrity number (RIN) value above 8 was used for following library preparation.

Next generation sequencing libraries were constructed according to the manufacturer’s protocol (NEBNext^®^ Ultra^TM^ RNA Library Prep Kit for Illumina^®^). Briefly, rRNA was removed using a Ribo-Zero-rRNA Removal Kit. Fragmentation was carried out using divalent cations under elevated temperature in NEBNext First Strand Synthesis Reaction Buffer (5X). First strand cDNA was synthesized using random hexamer primer and M-MuLV Reverse Transcriptase (RNase H-). Second strand cDNA synthesis was subsequently performed using DNA Polymerase I and RNase H. Remaining overhangs were converted into blunt ends via exonuclease/polymerase activities. After adenylation of 3′ ends of DNA fragments, NEBNext Adaptor with hairpin loop structure was ligated to prepare for hybridization. In order to select cDNA fragments of preferentially 150∼200 bp in length, the library fragments were purified with AMPure XP system (Beckman Coulter, Beverly, MA, United States). Then 3 μL USER Enzyme (NEB, United States) was used with size-selected, adaptor-ligated cDNA at 37°C for 15 min followed by 5 min at 95°C before PCR. Then PCR was performed using Phusion High-Fidelity DNA polymerase, Universal PCR primers and Index (X) Primer. At last, PCR products were purified (AMPure XP system) and library quality was assessed on the Agilent Bioanalyzer 2100 system.

The clustering of the index-coded samples was performed on a cBot Cluster Generation System using TruSeq PE Cluster Kit v3-cBot-HS (Illumina) according to the manufacturer’s instructions. After cluster generation, the library preparations were sequenced on an Illumina HiSeq 4000 platform. Sequencing was carried out using a 2 × 150 bp paired-end (PE) configuration, while image analysis and base calling were conducted by the HiSeq Control Software (HCS) + OLB + GAPipeline-1.6 (Illumina, CA, United States) on the HiSeq instrument.

### Transcriptomic Analysis

The raw mRNA sequence reads were pre-processed using a NGS QC Toolkit (*v.* 2.3) to remove low-quality bases with quality scores < 30 and adapter sequences (Patel and Jain, 2012). For paired-end Illumina reads, both pairs were removed if either pair mapped to rRNA sequences. Remaining reads were mapped to the *Synechocystis* genome which was downloaded from NCBI using Burrows-Wheeler Alignment tool software version (*v*. 0.7.5a) with default parameters (Li and Durbin, 2009). Raw counts of reads that uniquely mapped to each gene region were calculated by HTSeq (*v*. 0.6.1) (Anders et al., 2015). Then reads counts were normalized to the aligned FPKM (Fragments per kilobase of gene per million mapped fragments) to obtain the relative expression levels. Differential expression analysis between WT and *nc117* overexpression strains (WT-pJA2-*nc117*) was performed using the DESeq2 software (Anders and Huber, 2010), which used a model based on the negative binomial distribution. The resulting *p*-values were adjusted using the Benjamini and Hochberg’s approach for controlling the false discovery rate. Genes with fold change > 1.2 and adjusted *p*-values < 0.05 were assigned as differentially expressed.

The metabolic pathway analysis of the genes was conducted according to KEGG Pathway Database. The significance of whether differently expressed genes were enriched in a given functional pathway or functional category was calculated by the Wallenius non-central hypergeometric test using the GOseq *R* package in which gene-length bias was corrected (Young et al., 2010).

### qRT-PCR Validation

The RNA samples used in qRT-PCR were prepared from identical cultures for transcriptomic analysis above, and qRT-PCR analysis was performed as previously described (Wang et al., 2012). Quantification of mRNA expression was determined according to a standard process of qRT-PCR that used serial dilutions of known concentrations of chromosomal DNA as a template to construct a standard curve. A total of 20 genes were selected for validation and 16S rRNA was used as an internal control. Three technical replicates were analyzed for each sRNA. The data analysis was carried out using the StepOnePlus analytical software (Applied Biosystems, Foster City, CA, United States). Briefly, the amount of relative gene transcript was normalized by that of 16S rRNA in each sample, and the data presented were ratios of the amount of normalized transcripts in the treatment between the WT and mutant strains (All Primers were listed in **Supplementary Table S1**). Later, a *Pearson* correlation and statistically significant analysis with a set of transcripts between RNA-seq and qRT-PCR was conducted to ensure the reliability of RNA-seq by *R* software *corr.test* function.

## Results

### 3′ End Determination of sRNA Nc117

**FIGURE 1:**
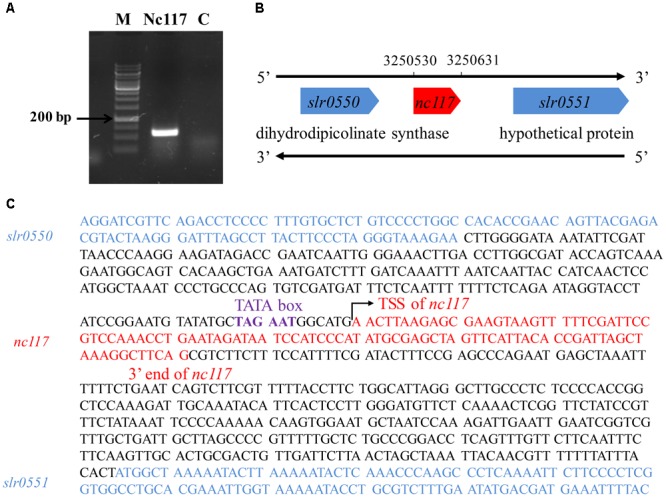
Determination of the origination and end of the sRNA Nc117. **(A)** 3′ ends of Nc117 orientation are determined by RACE. **(B)** Schematic presentation of Nc117. Number in the top DNA strands indicates the positions. **(C)** TSS and the 3′ end of Nc117 in the top DNA strands are indicated, respectively. The TATA box site is also indicated.

The *trans*-encoded sRNA Nc117 was demonstrated to be involved in the tolerance to biofuels ethanol and 1-butanol in *Synechocystis* in our previous study (Pei et al., 2017). However, its exact length remained to be elucidated as our previous study employed the length of Nc117 for overexpression only basing on the RNA-seq data (Pei et al., 2017). Previously, Nc117 has also been reported by Mitschke et al. (2011) (named as Ncr1600), in which the 5′ end of Nc117 has been determined while its 3′ end remained unclear. Here in this study we further determined the 3′ end of Nc117 employing 3′ rapid amplification of cDNA end (3′ RACE) technology (**Supplementary Figure S1**). As shown in **Figure 1A**, a distinct signal with fragment length of approximately 110 nucleotides was detected for Nc117. After Sanger sequencing, we pinpointed the 3′ end of Ncl17 at 3,250,631 of the *Synechocystis* genome, which was 16 bp shorter than that reported in our previous study (Pei et al., 2017). Thus, the length of Nc117 was determined to be 102 nucleotides, which was located between *slr0550* and a polycistron including *slr0551, slr0552*, and *slr0553* encoding three hypothetical proteins, as depicted in **Figure 1B**. Interestingly, the TATA box motif was also found right before the TSS of Nc117 (**Figure 1C**), suggesting the reliability of our analysis.

### Confirmation of the Involvement of Nc117 in Short Chain Alcohols Tolerance

**FIGURE 2:**
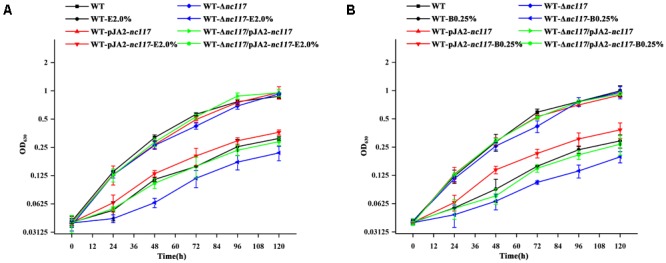
Growth curves of WT, *nc117* overexpression, *nc117* knockout and complementary strains under control and biofuels stress conditions. **(A)** WT, WT-pJA2-*nc117*, WT-Δ*nc117* and WT-Δ*nc117*/pJA2-*nc117* in normal BG11 medium with or without 2.0% (*v*/*v*) ethanol (E 2.0%). **(B)** WT, WT-pJA2-*nc117*, WT-Δ*nc117* and WT-Δ*nc117*/pJA2-*nc117* in normal BG11 medium with or without 0.25% (*v*/*v*) 1-butanol (B 0.25%). The error bar represents the calculated standard deviation of the measurements of three biological replicates.

To further confirm the involvement of Nc117 in short chain alcohols tolerance, a series of engineered strains were constructed in the *Synechocystis* WT based on the exact length of Nc117 determined above, including *nc117* overexpressing strain WT-pJA2-*nc117, nc117* knockout strain WT-Δ*nc117*, and the complementation strain WT-Δ*nc117*/pJA2-*nc117* by introducing the plasmid pJA2-*nc117* into the WT-Δ*nc117* mutant, and then their phenotypes under short chain alcohols stress were comparatively monitored. Consistent with the previous phenotypic observation (Pei et al., 2017), the growth of WT-Δ*nc117* was slower than WT under 2.0% (*v/v*) ethanol or 0.25% (*v/v*) 1-butanol conditions, while the growth of WT-pJA2-*nc117* was faster than the WT strain under the same conditions (**Figure 2**), indicating the direct involvement of Nc117 in short chain alcohols tolerance. In addition, the complementation of the sRNA *nc117* in the WT-Δ*nc117* mutant was able to bring the short chain alcohols tolerance to the similar level as the WT, demonstrating that the sRNA Nc117 was indeed involved positively in short chain alcohols tolerance in *Synechocystis*.

### Computational Target Prediction of Nc117 by IntaRNA

Based on the full length of the *trans*-encoded sRNA Nc117 determined in this study, target prediction was re-performed by IntaRNA software, and the top 100 predicted interaction targets were obtained (listed in **Supplementary Table S2**). Although the full sequence of Nc117 determined in this study had a few base pairs shifting compared to our previous analysis (Pei et al., 2017), more than half of the predicted target genes were identical to those reported in the previous study (Pei et al., 2017). According to the COG category classification, the functional pathways to which the predicted targets belonged included genes involved in replication, recombination and repair, cell wall/membrane/envelope biogenesis, amino acid transport and metabolism, and signal transduction mechanisms and so on (**Supplementary Table S2**). Besides, some genes predicted by IntaRNA have been reported involved in cyanobacterial tolerance to various kinds of environmental stresses previously. For example, *slr0415* encoding Na(+)/H(+) antiporter was found related to salt stress response and internal pH regulation of the *Synechocystis* (Elanskaya et al., 2002), and the Δ*sll0272* mutant was sensitive to heat stress for growth in *Synechocystis* (Gao F. et al., 2016). Interestingly, most of the interaction areas were located in the central region of the sRNA Nc117 (**Supplementary Figure S2**), indicating possible conserved functional binding domain of sRNA Nc117. Accordingly, for the possible targets mRNA of Nc117, most of the interaction domains were close to or overlapped with the upstream region of the target genes, especially with the RBSs or the translational start codon (**Supplementary Figure S3**), which was consistent well with the sRNA interaction mechanism reported in bacteria (Georg et al., 2014).

### Genome-Wide Identification of Potential Targets of Nc117 Based on Transcriptomic Analysis

**FIGURE 3:**
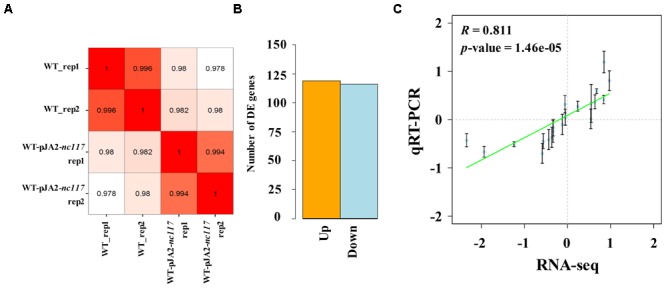
RNA-seq results analysis. **(A)** *Pearson* correlation analysis between all biological RNA-sample. **(B)** Differential expression analysis of RNA-seq. **(C)** Correlation between qRT-PCR and RNA-seq analyses for selected genes. For RNA-seq (horizontal coordinate), values represent log_2_ fold change of transcripts. For qRT-PCR (vertical coordinate), values represent the mean log_2_ fold change in transcripts. The error bar represents the standard deviation of three technical and three biological replicates. The *Pearson* correlation coefficients and their significances are indicated at the right lower corner of each plot.

Growth of *nc117* overexpression strain WT-pJA2-*nc117* was faster than the WT strain under short chain alcohols stress conditions (**Figure 2**). Then, RNA-sequencing (RNA-seq) based comparative transcriptomic analysis was conducted between WT and WT-pJA2-*nc117* strains to investigate the target genes regulated by Nc117. After RNA-seq and data filtering, a total of 63 million clean reads were generated in four samples between WT and WT-pJA2-*nc117* (each with two biological replicates). Subsequently, the qualified reads were mapped to the complete genome of *Synechocystis*, and all samples showed a mapping ratio greater than 96% (details in **Supplementary Table S3**). In addition, the *Pearson* correlation analysis between two biological replicates was greater than 0.99, indicating the reliability of our RNA-seq results (**Figure 3A**). To investigate target genes potentially regulated by sRNA Nc117, the differential expression profiling analysis was performed using DESeq2 software (Anders and Huber, 2010). Notably, a total of 235 genes were found differentially expressed in WT-pJA2-*nc117* compared with WT (**Supplementary Table S4**). Among them, 119 and 116 genes were up- and down-regulated, respectively (**Figure 3B**). To investigate the reliability of RNA-seq results, a subset of 20 target genes were then randomly selected for qRT-PCR confirmation. As shown in **Figure 3C**, the *Pearson* correlation analysis between qRT-PCR and RNA-seq data showed a clear positive correlation with correlation coefficients greater than 0.8 and *p*-value less than 0.005, suggesting the high reliability of RNA-seq results.

**FIGURE 4:**
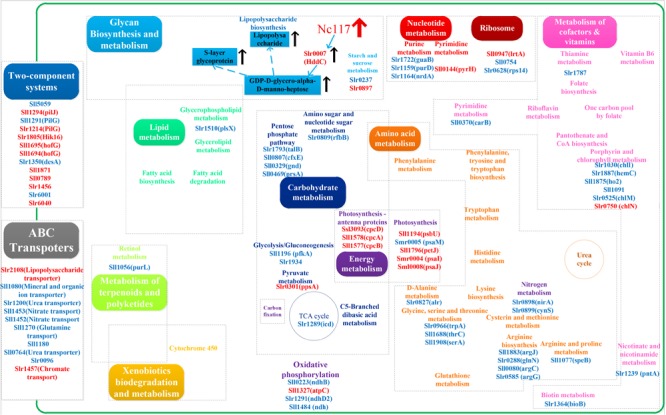
Scheme of global effects of sRNA Nc117 revealed by RNA-seq analysis. The metabolic features presented were deduced by RNA-seq analysis, and up-regulated genes (red) and down-regulated genes (blue) were labeled, respectively.

Interestingly, among the differentially regulated genes, some genes were also associated with cyanobacterial tolerance to various kinds of environmental stresses. For example, *slr0897* encoding β-1,3-1,4-glucanase functioning in salt stress tolerance in *Synechocystis* (Tamoi et al., 2007) was found up-regulated by 1.7-fold, while another gene *slr1667* regulated by a cAMP receptor protein involved in the construction of cell surface against ultraviolet radiation (Yoshimura et al., 2002, 2010), was up-regulated by 2.22-fold. The scheme of pathway mediated by sRNA Nc117 revealed by RNA-seq results was shown in **Figure 4**. Among them, the most differentially regulated pathways based on RNA-seq transcriptomics analysis were TCSTS and ABC transporters (**Figure 4**). These two pathways were both reported to be involved in regulating resistance under stress conditions (Los et al., 2010; Foo et al., 2014). For example, Uchiyama et al. (2012) found that *slr1214* encoding two-component response regulator PatA subfamily, may affect acid stress tolerance (Uchiyama et al., 2012). Although many differentially expressed genes were found encoding hypothetical proteins, the results showed that the up-regulated genes were significantly enriched in several functional categories, such as “photosynthesis”, “photosynthesis antenna proteins,” and “two-component system,” while most down-regulated genes were enriched in “pentose phosphate pathway” and “arginine and proline metabolism” (**Supplementary Table S5**).

### Identification of Direct Targets of Nc117 in Regulating Short Chain Alcohols Tolerance

**FIGURE 5:**
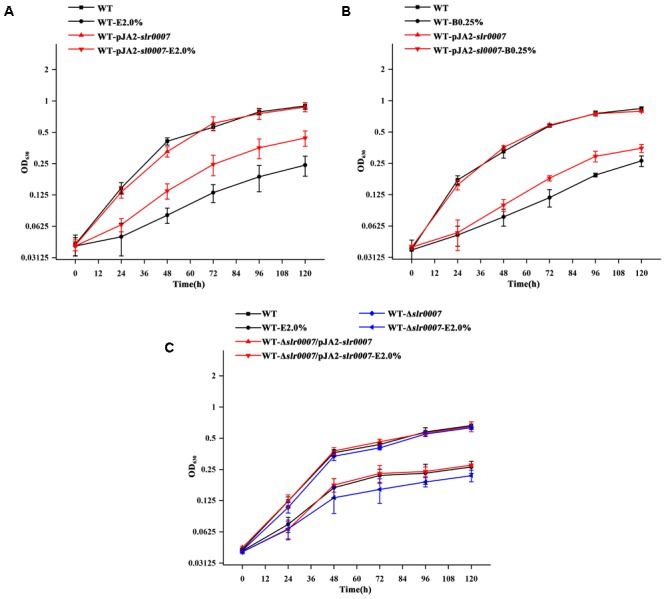
Growth curves of WT, overexpression, knockout and complementary strains under control and biofuels stress conditions. **(A)** WT, WT-pJA2-*slr0007* in normal BG11 medium with or without 2.0% (*v*/*v*) ethanol (E 2.0%). **(B)** WT, WT-pJA2-*slr0007* in normal BG11 medium with or without 0.25% (*v*/*v*) 1-butanol (B 0.25%). **(C)** WT, WT-Δ*slr0007* and WT-Δ*slr0007*/pJA2-*slr0007* in normal BG11 medium with or without 2.0% (*v*/*v*) ethanol (E 2.0%). The error bar represents the calculated standard deviation of the measurements of three biological replicates.

Considering a large set of putative target genes of Nc117 identified by comparative transcriptomic analysis, an integrative analysis combining transcriptomic analysis and computational target prediction was performed to narrow down the possible target genes of Nc117 that might be responsible for the short chain alcohols tolerance. We first conducted a comparative analysis of the top ranked computational predicted targets and the upstream regions of significantly differently expressed genes in comparative transcriptomic analysis. Interestingly, the upstream regions of five genes, i.e., *slr2108, slr0007, sll0784, sll1830*, and *slr1501* were identified overlapped. We then performed a qRT-PCR analysis for these five genes, and the results confirmed their differentially regulated expression in the *nc117* overexpression strain revealed by the transcriptomic analysis (**Table 2**). Therefore, these genes were selected for further functional characterization. Briefly, all the selected five genes were individually overexpressed and knockout, and their phenotypes under short chain alcohols stress were comparatively monitored in shake flasks. The results showed that one strain, the *slr0007* overexpressed strain, was found to be more resistant to ethanol and 1-butanol compared with WT (**Figures 5A,B**), and the knockout mutant Δ*slr0007* was more sensitive to ethanol compared with WT (**Figure 5C**), while there was no significant difference for the knockout mutants or overexpression strains of the other four genes compared with WT (data not shown). Furthermore, the complementation of *slr0007* in the Δ*slr007* mutant restored the ethanol tolerance to the level of WT (**Figure 5C**), indicating that *slr0007* was indeed involved in short chain alcohols tolerance positively regulated by Nc117.

**Table 2:** Possible target genes identified by target prediction and RNA-seq.

Gene	Fold change in RNA-seq analysis	Fold change in qRT-PCR analysis	Description
*slr1501*	0.30	0.77 ± 0.21	Probable acetyltransferase
*slr2108*	1.46	1.27 ± 0.23	Probable polysaccharide ABC transporter ATP binding subunit
*sll1830*	0.68	0.18 ± 0.15	Unknown protein
*slr0007*	1.33	1.46 ± 0.20	Probable sugar-phosphate nucleotidyltransferase
*sll0784*	0.42	0.43 ± 0.18	Nitrilase

**FIGURE 6:**
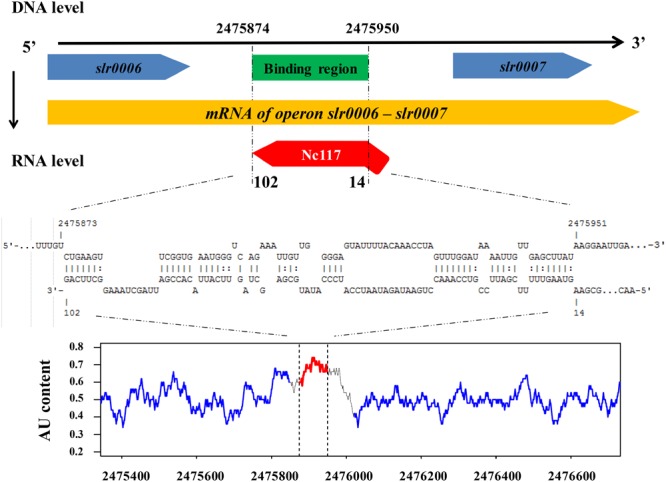
Interaction model of Nc117 and the target gene. The upper part shows the location and interact region between Nc117 and *slr0006*-*slr0007* operon in DNA level and RNA level. The lower part indicated the AU content (box in 50 bp) of *slr0006*-*slr0007* operon, red represents interact region and blue represents gene *slr0006* **(left)** and *slr0007* **(right)** coding sequence.

According to KEGG pathway database, *slr0007* encodes the HddC involved in the LPS synthesis pathway, which might contribute to the short chain alcohols tolerance regulation (Leone et al., 2007). According to the comparative transcriptomic analysis above, the mRNA level of *slr0007* was up-regulated 1.33-fold in the WT-pJA2-*nc117* strain (**Table 2**). Concerning the mechanism of sRNA Nc117 in the regulation of *slr0007*, Nc117 may either enhance the *de novo* synthesis of *slr0007* mRNA or suppress the degradation of *slr0007* mRNA. To investigate whether the increased transcript accumulation of *slr0007* in WT-pJA2-*nc117* strain was due to transcriptional activation or post-transcriptional stabilization, a further bioinformatics analysis to predict the interaction between Nc117 and *slr0007* together with its upstream region was firstly performed. As shown in **Figure 6**, the interaction region between Nc117 and *slr0007* was predicted to be located in the upstream of *slr0007* (intergenic region between *slr0006* and *slr0007*). According to the previous RNA-seq analysis by Kopf et al. (2014), *slr0006* and *slr0007* composed an operon (Kopf et al., 2014), suggesting the interaction region between Nc117 and *slr0007* might not be TSS or promoter region. However, according to a previous study by Pfeiffer et al. (2009), despite sRNAs could make base pair with the 5′ UTR of mRNA targets, some sRNAs such as MicC were shown to function with target mRNA by recognition of their mRNA molecule, guiding RNase E to cleave inside the coding sequence (Pfeiffer et al., 2009). Interestingly, our comparative transcriptomic results showed that the expression level of *slr0006* was also 1.21-fold up-regulated in WT-pJA2-*nc117* strain compared with WT. In addition, the AU content in the intergenic region between *slr0006* and *slr0007* was significantly higher than their coding sequence region, as shown in **Figure 6**, which is typical characteristic of a potential target cleavage site of RNase E in cyanobacteria (Horie et al., 2007). All these findings pointed to a hypothesis that the up-regulation of *slr0006* and *slr0007* in Nc117 overexpression strain was due to the protection role of Nc117 in protecting mRNA molecule of operon *slr0006* and *slr0007*, although further evidences are still needed.

## Discussion

As regulatory elements to deal with stress conditions in prokaryotes, sRNAs have received considerable attention in recent years. To understand the regulation mechanism of sRNAs, the primary targets should first be identified. So far, a series of methods for determining sRNAs target genes were developed (Vogel and Wagner, 2007). Among them, computational target prediction was demonstrated to be one of the effective approaches (Busch et al., 2008). In this study, the computational target prediction was first employed to explore potential targets of Nc117, and the top 100 potential targets were obtained. Although only 5 targets were demonstrated to be overlapped with those identified by the following RNA-seq transcriptomic analysis, considering the possibility that Nc117 might be involved in other functions besides short chain alcohols resistance, we can’t fully exclude some of these predicted targets as potential targets of Nc117, which remained to be investigated. Meanwhile, RNA-seq based comparative transcriptomic analysis of *nc117* overexpression strain WT-pJA2-*nc117* and WT revealed that 119 and 116 genes were up- and down-regulated, respectively, although most of these regulated genes may be indirect. By functional characterization of overexpression and knockout of the overlapped 5 potential targets, only *slr0007* was demonstrated to be the potential target of Nc117 in regulating short chain alcohols tolerance (**Figure 5**). For the other four genes (*slr2108, sll0784, sll1830*, and *slr1501*), the similar phenotype in alcohols resistance was not observed as *slr0007*, which might be due to several possible reasons: (i) the significance of involvement in alcohols tolerance: these genes may be involved in alcohols tolerance, but we failed to identify them under the tested conditions; (ii) the changes of these genes may be secondary responses of genes regulated by Nc117, or be involved in other functions instead of short chain alcohols resistance that are still yet to be investigated; (iii) the false positive from the deficiency of computational target prediction and RNA-seq transcriptomic analysis. To further get more reliable target genes, more analysis such as comparative proteomics analysis can be employed.

The gene *slr0007* was demonstrated to be the potential target of Nc117 in regulating short chain alcohols tolerance. According to an early conclusion by Cuccui et al. (2012), most Gram-negative bacteria tended to have higher organic solvent tolerance than Gram-positive bacteria, and it was speculated that the extra outer membrane structure unique to Gram-negative bacteria may be one of the key reasons for tolerance differences. LPS, as a main component of the outer membrane of Gram-negative bacteria, helped maintain the integrity of the outer membrane structure and was the first barrier to resist external environmental stress (Snyder et al., 2009). It prevents not only the dissolution of complement, antimicrobial peptide and detergent, but also the penetration of harmful substances to some extent (Trautner and Vermaas, 2013). According to Park et al. (2017), the carbohydrate glycero-manno-heptose and its derivatives were crucial parts of LPS in most Gram-negative bacteria, and they existed widely in core oligosaccharide region, O antigens and even glycan chains of S-layer glycoproteins. The target identified in this study, HddC encoded by *slr0007* was one of the essential enzymes for the synthesis of carbohydrate glycero-manno-heptose (Cuccui et al., 2012). In addition, HddC may be related to the synthesis of surface layer glycoprotein, as an early study by Trautner and Vermaas (2013) showed that deletion of S-layer glycoprotein in *Synechocystis* led to low survival ability when exposed to lysozyme treatment or under low osmotic pressure, as well as the protective effect of the S-layer glycoprotein on the cyanobacterium (Trautner and Vermaas, 2013). In this respect, our results pointed to the proposed regulation mechanism of Nc117 that it may directly regulate synthesis of LPS or S-layer glycoprotein, correspondingly affecting tolerance to short chain alcohols. On the other hand, *trans*-encoded sRNAs usually regulate mRNAs by short, imperfect base pairing interactions. Some of these sRNAs interaction base pairs are at or near the 5′ UTR of their targets, and other family members interaction base pair may be at more distant sites (Storz et al., 2011). They have positive or negative regulatory effects on the expression of the corresponding genes by influencing translation or changing the stability of mRNA. Although it was hypothesized that the up-regulation of *slr0006* and *slr0007* in Nc117 overexpression strain was due to the protection role of Nc117 in protecting mRNA molecule of operon *slr0006* and *slr0007*, further evidences are also still needed. Nevertheless, the identification of the involvement of *slr0007* in alcohols tolerance helped to better understand the tolerance regulation mechanism to alcohols in cyanobacteria, and could be used to further rationally design and construct more tolerant cyanobacterial chassis, for example, by gene mutation to gain a protein with improved activity or other synthetic biology approaches, in the future.

## Conclusion

sRNAs are important regulatory elements for coping with stress in prokaryotes. In this study, functional characterization of Nc117 in regulating biofuels tolerance in *Synechocystis* was conducted. By employing computational target prediction, the top 100 target genes were obtained. In addition, by comparative transcriptomic RNA-seq analysis, a total of 119 up-regulated and 116 down-regulated genes were identified. Integration of both analysis allowed us narrow down the possible targets to five for which the upstream regions of 5 genes were overlapped with those identified by computational target prediction approach. Notably, further functional characterization using overexpression and knockout strains showed that one target gene *slr0007* was directly regulated by Nc117 involved in short chain alcohols tolerance. The results suggested that Nc117 was involved in short chain alcohols tolerance by regulating *slr0007* and synthesis of LPS or S-layer glycoprotein. This study not only provided a novel regulatory mechanism of *trans*-encoded sRNA in cyanobacteria, but also valuable information for high-tolerant chassis construction in photosynthetic cyanobacteria.

## Additional Information

The raw mRNA sequence data of *Synechocystis* are deposited in the SRA database of NCBI with accession numbers SRP130885.

## Author Contributions

GP, LC, and WZ conceived the study. GP executed the sRNA and transcriptome bioinformatic analysis. YB, GP, and ZC carried out the mutant construction, phenotypic analysis, qRT-PCR experiments, and determination of RNA half-lives. TS and GP performed the 3′ RACE. YB, GP, TS, LC, and WZ designed and revised this paper. All authors read and approved the final manuscript.

## Conflict of Interest Statement

The authors declare that the research was conducted in the absence of any commercial or financial relationships that could be construed as a potential conflict of interest.

## Supplementary Material

FIGURE S1 3′ end determination of sRNA Nc117 by 3′ RACE and previous 5′ origination location.

FIGURE S2 Predicted interaction region in sRNA.

FIGURE S3 Predicted interaction region in mRNA.

TABLE S1 Primers used for qRT-PCR validation.

TABLE S2 Targets information for Nc117 based on IntaRNA prediction.

TABLE S3 RNA-seq mapping statistics of different samples.

TABLE S4 RNA-seq analysis of wild type *Synechocystis* and *nc117* overexpression strain.

TABLE S5 Pathway enrichment analysis for differential expressed genes.

## Abbreviations

3′ RACE: 3′ rapid amplification of complementary DNA end
cDNA: complementary DNA
HddC: D-glycero-alpha-D-manno-heptose 1-phosphate guanylyltransferase
KEGG: Kyoto Encyclopedia of Genes and Genomes
LPS: lipopolysaccharide
qRT-PCR: quantitative real-time polymerase chain reaction
RBS: ribosomal binding site
RNA-seq: RNA sequencing
sRNA: small RNA
TCSTS: two-component signal transduction systems
TSS: transcriptional starting site
UTR: untranslated region
WT: wild type
