# Supplementary material for: Regulation Mechanism Mediated by Trans-Encoded sRNA Nc117 in Short Chain Alcohols Tolerance in Synechocystis sp. PCC 6803
Source: Front Microbiol. 2018 May 1;9:863. doi: 10.3389/fmicb.2018.00863 (PMC5946031; doi:10.3389/fmicb.2018.00863)
Supplement: FIGURE S1 — 3′ end determination of sRNA Nc117 by 3′ RACE and previous 5′ origination location. [file Presentation_1.PPTX]

## Slide 1
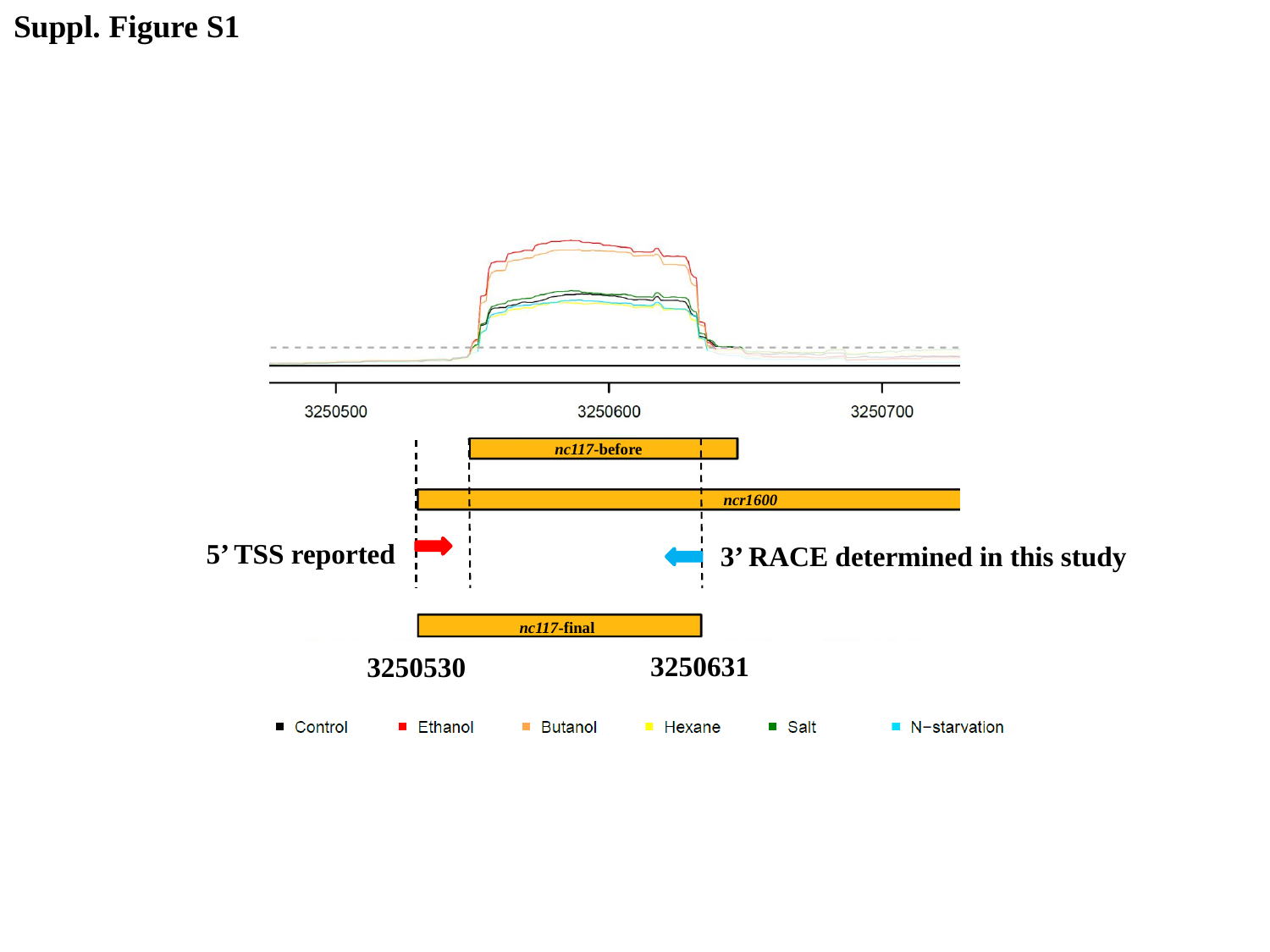

Suppl. Figure S1
nc117-before
ncr1600
5’ TSS reported
3’ RACE determined in this study
nc117-final
3250631
3250530

## Slide 2
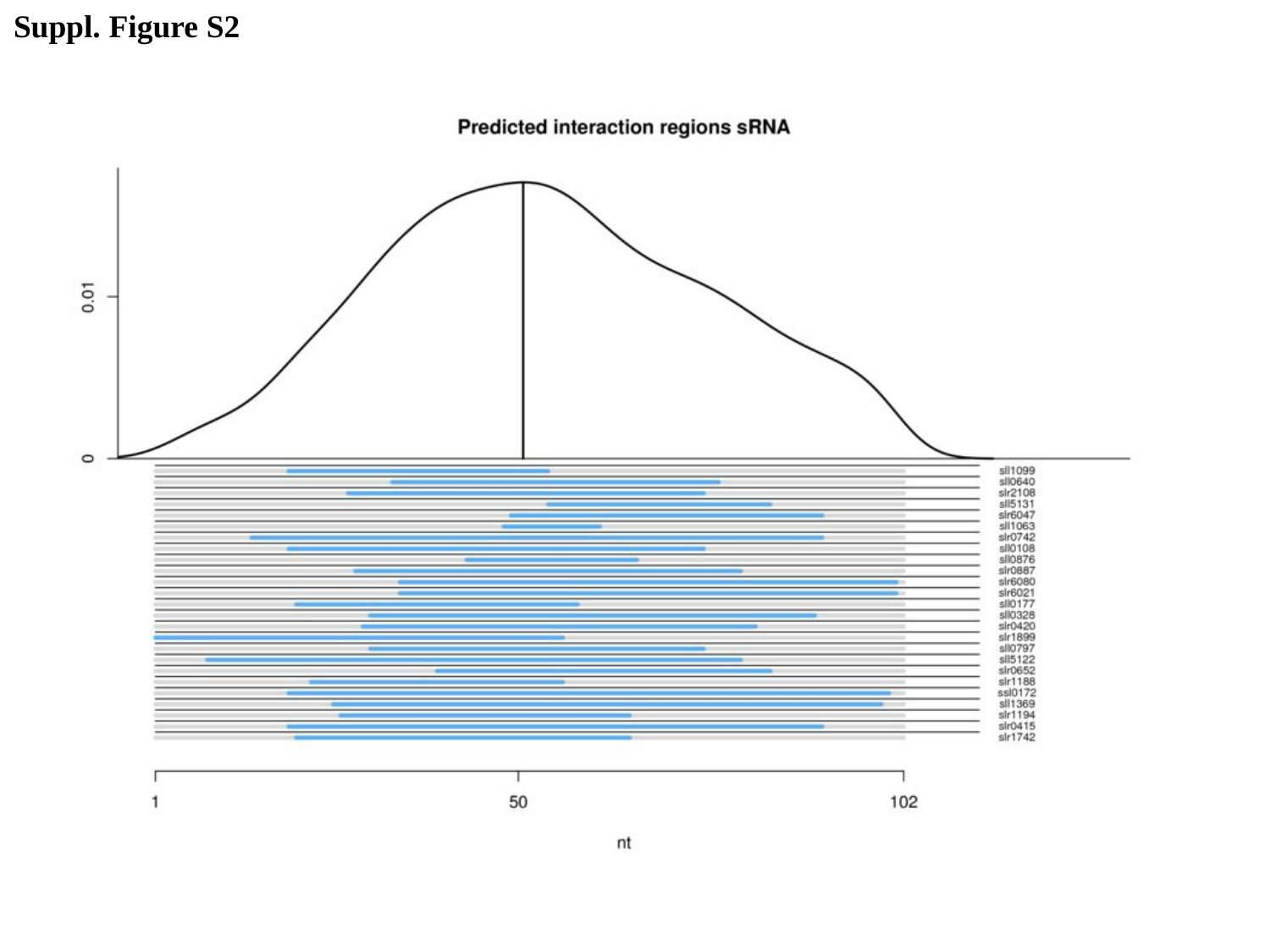

Suppl. Figure S2

## Slide 3
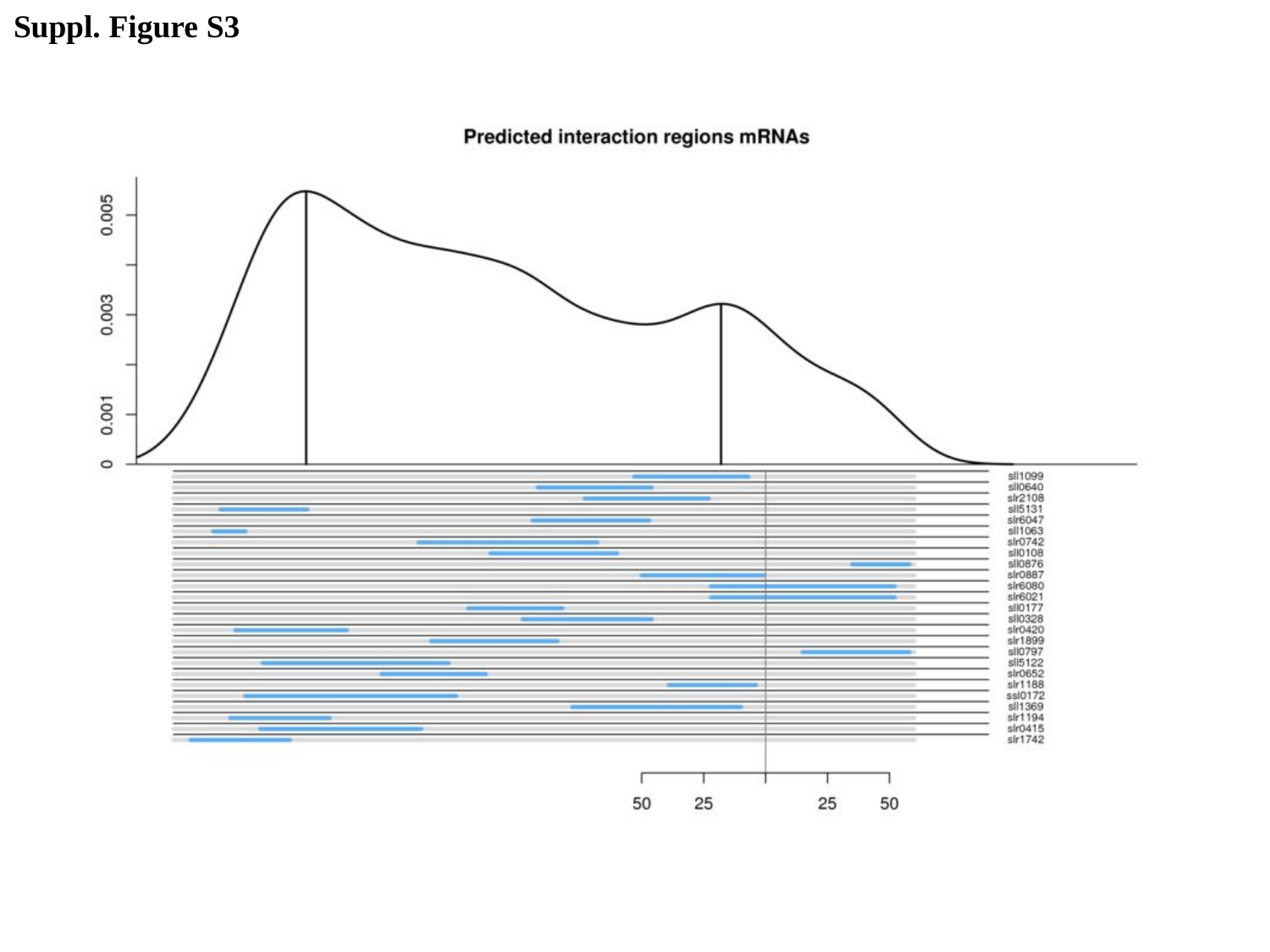

Suppl. Figure S3
